# Cyclodextrins Can Entrap Zearalenone-14-Glucoside: Interaction of the Masked Mycotoxin with Cyclodextrins and Cyclodextrin Bead Polymer

**DOI:** 10.3390/biom9080354

**Published:** 2019-08-09

**Authors:** Zelma Faisal, Eszter Fliszár-Nyúl, Luca Dellafiora, Gianni Galaverna, Chiara Dall’Asta, Beáta Lemli, Sándor Kunsági-Máté, Lajos Szente, Miklós Poór

**Affiliations:** 1 Department of Pharmacology, Faculty of Pharmacy, University of Pécs, Szigeti út 12, H-7624 Pécs, Hungary; 2 János Szentágothai Research Center, University of Pécs, Ifjúság útja 20, H-7624 Pécs, Hungary; 3 Department of Food and Drug, University of Parma, Via G.P. 7 Usberti 17/A, 43124 Parma, Italy; 4 Institute of Organic and Medicinal Chemistry, Medical School, University of Pécs, Szigeti út 12, H-7624 Pécs, Hungary; 5 CycloLab Cyclodextrin Research & Development Laboratory, Ltd., Illatos út 7, H-1097 Budapest, Hungary

**Keywords:** zearalenone-14-glucoside, masked mycotoxin, cyclodextrins, host–guest interaction, fluorescence spectroscopy, cyclodextrin bead polymer, mycotoxin binder, toxin removal

## Abstract

Zearalenone (ZEN) is a *Fusarium*-derived xenoestrogenic mycotoxin. In plants, zearalenone-14-*O*-β-d-glucoside (Z14G) is the major conjugated metabolite of ZEN, and is a masked mycotoxin. Masked mycotoxins are plant-modified derivatives, which are not routinely screened in food and feed samples. Cyclodextrins (CDs) are cyclic oligosaccharides built up from D-glucopyranose units. CDs can form stable host–guest type complexes with lipophilic molecules (e.g., with some mycotoxins). In this study, the interaction of Z14G with native and chemically modified β- and γ-CDs was examined employing fluorescence spectroscopy and molecular modeling. Furthermore, the removal of Z14G from aqueous solution by insoluble β-CD bead polymer (BBP) was also tested. Our results demonstrate that Z14G forms the most stable complexes with γ-CDs under acidic and neutral conditions (*K* ≈ 10^3^ L/mol). Among the CDs tested, randomly methylated γ-CD induced the highest increase in the fluorescence of Z14G (7.1-fold) and formed the most stable complexes with the mycotoxin (*K* = 2 × 10^3^ L/mol). Furthermore, BBP considerably reduced the Z14G content of aqueous solution. Based on these observations, CD technology seems a promising tool to improve the fluorescence analytical detection of Z14G and to discover new mycotoxin binders which can also remove masked mycotoxins (e.g., Z14G).

## 1. Introduction

Mycotoxins are toxic secondary metabolites of filamentous fungi, occurring in several food products (e.g., cereals, meat, fruits, and numerous beverages) [1]. Due to the consumption of contaminated food, mycotoxin exposure induces health problems in both animals and humans [2]. Zearalenone (ZEN) is a *Fusarium*-derived mycotoxin; it appears in cereals (e.g., in maize), beer, milk, spices, etc. [3,4]. Despite its non-steroidal structure, ZEN can cause reproductive disorders in animals and humans, due to its xenoestrogenic effect [5,6,7]. Furthermore, ZEN is a strongly phytotoxic compound, a protonophoric uncoupler, and it alters the permeability of some plant tissues [8,9]. Plants possess detoxification system, which protects them from harmful xenobiotics, including mycotoxins [10,11,12]. The formation of hydrophilic conjugates of mycotoxins is a common detoxification process in plants [11,12], such as the glucose conjugation of ZEN, from which zearalenone-14-*O-*β-d-glucoside is the main product (Z14G; also known as zearalenone-4-glucoside) [11,13]. These mycotoxin derivatives also appear in food, and after their oral consumption, they can be converted into the original mycotoxin (mainly by the colonic microbiota) [13]. The conversion of the conjugated mycotoxins into the more toxic parent mycotoxin increases the toxicological risk of exposure to the mycotoxin-contaminated food [14]. Since their determination and quantification is challenging, these plant-derived conjugates are not routinely analyzed in food samples [11,14,15]. Based on the systematic definition of Rychlik et al. [16], the plant-conjugated mycotoxins are known as “masked mycotoxins”, due to the difficulty of their analytical determination.

**Figure 1:**
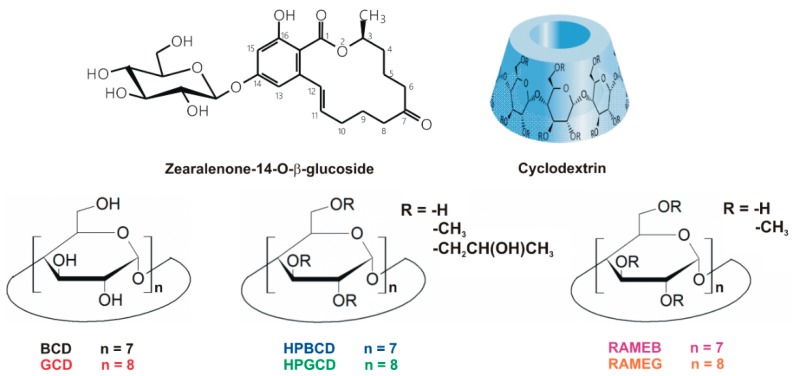
Chemical structures of zearalenone-14-*O-*β-d-glucoside (Z14G) as well as native, randomly methylated (DS = 12), and hydroxypropyl (DS = 4.5) β- and γ-CDs (BCD, β-cyclodextrin; GCD, γ-cyclodextrin; HPBCD, (2-hydroxypropyl)-β-cyclodextrin; HPGCD, (2-hydroxypropyl)-γ-cyclodextrin; RAMEB, randomly methylated β-cyclodextrin; RAMEG, randomly methylated γ-cyclodextrin; DS: average degree of substitution per CD ring).

Z14G (Figure 1) is one of the few proven, naturally occurring masked mycotoxins in cereals (e.g., wheat, maize, and barley) [11]. During the biotransformation of ZEN in plants, several metabolites are formed, Z14G accounts for up to 30% of the total metabolites [11]. The glucose conjugation of the parent compound leads to its decreased estrogenicity, because the lactone ring and the hydroxyl group in position C_14_ are involved in the interaction of ZEN with estrogen receptors [14,17]. However, Z14G is deglycosylated in the gastrointestinal tract, and the formed ZEN can induce toxic effects due to its better absorption and higher affinity towards estrogen receptors [18]. In addition, it has also been proved that cell metabolism may convert Z14G to ZEN and α-ZEL eliciting estrogenic stimulation [19].

Cyclodextrins (CD) are starch-derived oligosaccharides, built up by d-glucopyranose units [20]. The most frequently used CDs are α-, β-, and γ-CDs containing six, seven, and eight glucose units, respectively. CDs are commonly applied in the pharmaceutical, cosmetic, and food industries, due to their ability to form host–guest type complexes with several compounds [21]. CDs have a nonpolar internal cavity, and a hydrophilic external surface. The apolar cavity can accommodate lipophilic guest molecules, while the hydrophilic external part ensures excellent aqueous solubility [20,22]. The chemical modification of CDs can significantly affect their interaction with the guest molecules [20].

CDs can form stable complexes with some mycotoxins, including aflatoxins, citrinin, ochratoxin A, ZEN, and zearalenols [23,24,25,26,27,28,29]. The inclusion of these fluorescent mycotoxins by the CD cavity is commonly useful in analytics, because it can improve their chromatographic properties and/or increase their fluorescence signal [30,31,32]. Furthermore, previous studies demonstrated that CD polymers may be useful as mycotoxin binders: the extraction of ZEN, zearalenols, ochratoxin A, and patulin mycotoxins was successfully executed from aqueous solutions and from different beverages [33,34,35]. Despite masked mycotoxins (e.g., Z14G) also appearing in food products, we have no information regarding the potential interactions of CDs with these mycotoxin derivatives. ZEN forms stable complexes with CDs (*K*~10^4^ L/mol) [25,27]; however, Z14G contains the large hydrophilic glucose structure, which does not make obvious the interaction of the masked mycotoxin with CDs.

In this study, the interaction of Z14G with native and chemically modified β- and γ-CDs (Figure 1) was investigated in a wide pH range (pH 3.0–10.0) employing steady-state fluorescence spectroscopy. In addition to the stability of the complexes formed, the CD-induced increase in the fluorescence signal of Z14G was also evaluated. For the deeper understanding of Z14G-CD interactions, molecular modeling studies were performed. Furthermore, the removal of Z14G from aqueous solution by insoluble β-cyclodextrin bead polymer (BBP) was also tested. Our results demonstrate that Z14G can form stable host–guest type complexes with CDs, and the interactions result in the strong increase in the fluorescence of the mycotoxin. Moreover, BBP significantly decreased the Z14G content of the spiked solution, showing that CD technology is suitable for the removal of the masked mycotoxin Z14G from aqueous solutions.

## 2. Materials and Methods

### 2.1. Reagents

Zearalenone-14-*O*-β-d-glucoside (Z14G) was purchased from ASCA GmbH (Berlin, Germany). Stock solutions of Z14G (5000 µM) were prepared in ethanol (96 *v/v*%, spectroscopic grade; Reanal, Budapest, Hungary) and stored at −20 °C. Cyclodextrins, including β-CD (BCD), γ-CD (GCD), (2-hydroxypropyl)-β-CD (HPBCD), (2-hydroxypropyl)-γ-CD (HPGCD), randomly methylated β-CD (RAMEB), randomly methylated γ-CD (RAMEG), and insoluble β-cyclodextrin bead polymer (BBP) were provided by CycloLab Cyclodextrin Research and Development Laboratory, Ltd. (Budapest, Hungary). Sodium phosphate (0.05 M, pH 3.0 and pH 7.4), sodium acetate (0.05 M, pH 5.0), and sodium borate (0.05 M, pH 10.0) buffers were applied as media during fluorescence spectroscopic measurements.

BBP was produced in three main steps: (1) Pre-polymerization of monomeric BCD by cross-linking it with epichlorohydrin under alkaline circumstances. (2) Forming emulsion from the pre-polymer: the pre-polymer was emulsified in toluene-polyvinyl alcohol system with vigorous stirring. (3) Further polymerization: the emulsified BCD pre-polymer is further polymerized with butanediol bis(epoxypropyl)ether. During the latter step, the water-soluble pre-polymer became water-insoluble. The formed polymer droplets were filtered from the reaction mixture, washed with acetone, and dried. BBP does not dissolve but it swells in water (swelling capacity: 5–8 mL/g at 25 °C). The BCD content of BBP is 50 *m/m*%; the average polymer bead particle size is between 0.1 and 0.3 mm.

### 2.2. Fluorescence Spectroscopic Measurements

Steady-state fluorescence spectroscopic measurements were carried out at +25 °C, in the presence of air, using a Hitachi F-4500 fluorimeter (Hitachi, Tokyo, Japan). Fluorescence emission spectra of Z14G (1 µM) was recorded in the absence and presence of increasing concentrations of CDs (0.0, 0.2, 0.3, 0.5, 0.7, 1.0, 1.5, and 2.0 mM) in different buffers (pH 3.0–10.0; see 2.1), applying 315 nm excitation wavelength. Binding constants of Z14G-CD complexes were determined employing the graphical application of the Benesi-Hildebrand equation [26]:

(1) $$\frac{I_{0}}{\left(I-I_{0}\right)}=\frac{1}{A}+\frac{1}{A\times K\times {\left[H\right]}^{n}}$$

where *I*_0_ and *I* denote the fluorescence emission intensities of Z14G in the absence and presence of CDs, respectively; *A* is a constant, *K* is the binding constant (unit: L/mol), *[H]* is the concentration of the host molecule, and *n* is the number of binding sites.

### 2.3. Modeling Studies

The molecular modeling approach relied on a combination of pharmacophoric analysis of CD cavity, docking studies, and rescoring procedures. The 3D structures of BCD and GCD derived from the crystallographic structures recorded in the Cambridge Crystallographic Data Center (CCDC) database (https://www.ccdc.cam.ac.uk/structures) having accession code WEWTOJ and LAJLALO2, respectively. The ideal 3D coordinates of ZEN was retrieved from the Protein DataBank (https://www.rcsb.org; compound accession ID: ZER) [36]. The consistency of atom and bond types assignments were checked with the Sybyl software (version 8.1; www.certara.com) and the structure was energetically minimized using Powel algorithm, as described [37]. As an exception, the maximum number of iterations was set at 250 with a coverage gradient of ≤ 0.05 kcal/ (mol × Å). The 3D structure of Z14G was derived from editing the 3D structure of ZEN using the Sybyl software (version 8.1; www.certara.com) as follow: the 3D coordinates of glucose was retrieved using the “Get Fragment” module. Then, its hydroxyl group in position C_1_ was joined to the ZEN hydroxyl group on C_14_ using the “Join Molecule” option. The structure of Z14G finally underwent energy minimization.

#### 2.3.1. Pharmacophoric Analysis of the CD Cavity

The description of CD sites was done using the Flapsite tool of the FLAP software (Fingerprint for Ligand and Protein; https://www.moldiscovery.com), while the GRID algorithm was used to investigate the corresponding pharmacophoric space [38,39] in agreement with our previous study [40]. As an exception, it was used only the DRY probe to describe hydrophobic space of CD cavities.

#### 2.3.2. Docking Study

The GOLD software [41] was used to perform all the docking simulations as it previously proved to be reliable in predicting the binding architectures of host–guest complexes [42,43]. In addition, a rescoring procedure using the HINT scoring function [44] was carried out, for the better evaluation of mycotoxin–CD interactions [45]. In particular, the HINT score may be related to the free energy of binding (the higher the score, the stronger the interaction), and it was previously proved to assess reliably the host–guest type complex formation, also in the specific case of mycotoxin–CD interactions [45,46,47]. The GOLD setting reported by Dellafiora and co-workers was used [42]. Ten poses were generated for each compound in each CD, and all of them underwent a rescoring procedure with HINT. Only the best-scored pose for each run was considered [45,46].

### 2.4. Extraction of Z14G from Aqueous Solution by BBP

To test the mycotoxin binding ability of BBP, Z14G (2 μM, 1.5 mL) was incubated in the presence of increasing amounts of BBP (0.0, 1.0, 2.5, 5.0, 10.0, and 20.0 mg/1.5 mL). The incubation was performed in a thermomixer (1000 rpm, 30 min, 25 °C) in 0.05 M sodium acetate buffer (pH 5.0). Thereafter, BBP was sedimented by pulse centrifugation (4000 g, 3 s), and the concentration of Z14G in the supernatant was directly determined by HPLC-FLD (see details in Section 2.5). 

For the quantitative characterization of the interaction, Langmuir and Freundlich isotherms were also obtained. Using the same experimental conditions, increasing concentrations of Z14G (0.2, 0.5, 1.0, 2.5, 5.0, 7.5, 10.0, and 12.5 μM in 1.5 mL buffer) were added to a standard amount of BBP (2.5 mg). The evaluation was performed using the Langmuir equation [35]:

(2) $$q_{e}=\frac{\left(Q_{0}\times K_{L}\times C_{e}\right)}{\left(1+K_{L}\times C_{e}\right)}$$

where *q_e_* represents the bound Z14G (mg) per BBP (g), *Q*_0_ is the maximum amount of Z14G bound per g of BBP, *C_e_* denotes the free Z14G (mg) in the solution at equilibrium, and *K_L_* is the Langmuir equilibrium constant (L/mg). Data were then also evaluated based on the Freundlich equation [35]:

(3) $$q_{e}=K_{F}\times C_{e}^{1/n}$$

where *K_F_* and *n* are the Freundlich constant and the heterogeneity index, respectively.

### 2.5. HPLC Analysis

The concentrations of Z14G in the supernatants were quantified by an integrated HPLC system (Jasco, Tokyo, Japan) contained an autosampler (AS-4050), a binary pump (PU-4180), and a fluorescence detector (FP-920). Samples (with a 20-μL injected volume) were driven through a Phenomenex Security Guard™ (C8, 4.0 × 3.0 mm) guard column linked to a Teknokroma Mediterranea Sea8 (C8, 150 × 4.6 mm, 5 μm) analytical column. The mobile phase contained acetonitrile and 175 mM acetic acid (35:65 *v/v*%), the isocratic elution was performed with 1.0 mL/min flow rate at room temperature. Z14G was detected at 465 nm (λ_ex_ = 315 nm), and the chromatographic data were evaluated employing ChromNAV (V2) software.

### 2.6. Statistical Analyses

Data represent means ± standard error of the mean (SEM) values determined based on at least three independent experiments. The One-Way ANOVA test (IBM SPSS Statistics, V21, New York, NY, USA) was applied for the statistical analyses. The level of significance was set as *p* < 0.01. 

## 3. Results

### 3.1. Fluorescence Excitation and Emission Spectra of Z14G

**Figure 2:**
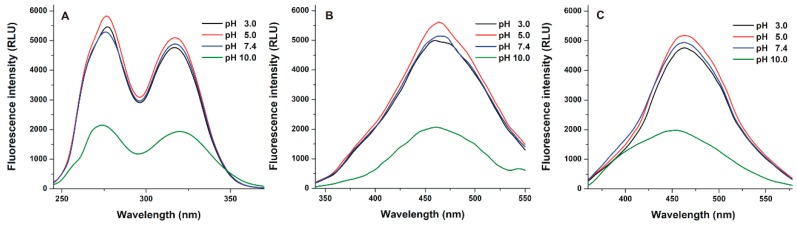
Fluorescence spectra of Z14G. (**A**) Excitation spectra of Z14G (20 µM) in different buffers (λ_em_ = 455 nm); (**B**) emission spectra of Z14G (20 µM) using 275 nm excitation wavelength; (**C**) and emission spectra of Z14G (20 µM) applying 315 nm excitation wavelength. (Buffers used: 0.05 M sodium phosphate, pH 3.0; 0.05 M sodium acetate, pH 5.0; 0.05 M sodium phosphate, pH 7.4; 0.05 M sodium borate, pH 10.0).

To investigate the effects of the environmental pH on the fluorescence of Z14G, its fluorescence excitation and emission spectra were recorded in different buffers (pH 3.0–10.0). At each pH value tested, two peaks appeared in the fluorescence excitation spectrum of Z14G, approximately at 275 and 315 nm (Figure 2A). At pH 3.0–7.4, the excitation spectra of Z14G barely changed; however, at pH 10.0, the significantly lower excitation signal of Z14G was observed compared to the other buffers. Then the fluorescence emission spectra of Z14G were also recorded using both 275 (Figure 2B) and 315 nm (Figure 2C) excitation wavelengths. Again, at acidic and physiological pH, similar emission spectra were observed. Nevertheless, the strong decrease in the fluorescence emission signal of Z14G was noticed under alkaline conditions. Furthermore, as Figure 2B,C demonstrates, a blue shift of emission maxima was observed with regard to both excitation wavelengths used (λ_ex_ = 275 nm: from 465 to 460 nm; λ_ex_ = 315 nm: from 465 to 450 nm).

### 3.2. Effects of Cyclodextrins on the Fluorescence Signal of Z14G 

**Figure 3:**
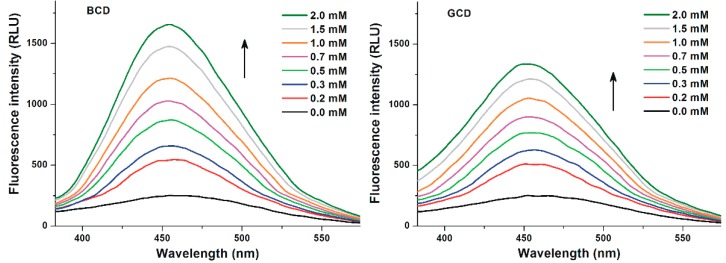
Fluorescence emission spectra of Z14G (1 µM) in the presence of increasing BCD (left) and GCD (right) concentrations (0.0–2.0 mM) in 0.05 M sodium acetate buffer (pH 5.0; λ_ex_ = 315 nm).

First, the interactions of Z14G with native BCD and GCD were investigated. Therefore, increasing amounts of CDs (final concentrations: 0.0–2.0 mM) were added to Z14G (1.0 μM) in sodium acetate buffer (pH 5.0), and then fluorescence emission spectra were recorded (λ_ex_ = 315 nm). Both BCD and GCD strongly increased the emission signal of the mycotoxin (Figure 3), during which a slight blue shift of its emission wavelength maximum (from 465 to 455 nm) was observed. Furthermore, BCD induced a higher increase in the fluorescence signal of Z14G than GCD. 

**Figure 4:**
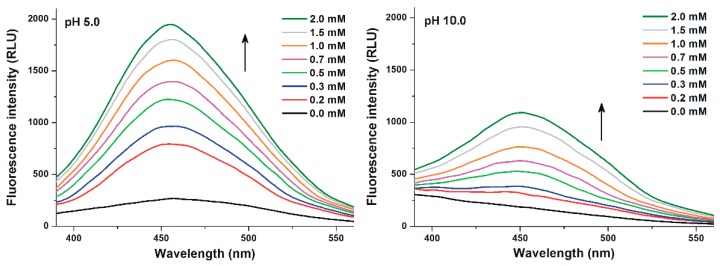
Fluorescence emission spectra of Z14G (1 µM) in the presence of increasing concentrations of RAMEG (0.0–2.0 mM) in 0.05 M sodium acetate (pH 5.0; left) and in 0.05 M sodium borate (pH 10.0; right) buffers (λ_ex_ = 315 nm).

Thereafter, the spectral changes of Z14G were also investigated with native and chemically modified CDs (methyl and hydroxypropyl derivatives) in different buffers (pH 3.0–10.0). At pH 10.0, a much lower increase in the fluorescence of the mycotoxin was observed compared with other buffers used (Figure 4). Furthermore, a red shift in the fluorescence spectrum of Z14G was observed at pH 10.0, resulting in the same emission wavelength maximum (455 nm) in the presence of higher CD concentrations which was noticed in other buffers with lower pH values.

**Figure 5:**
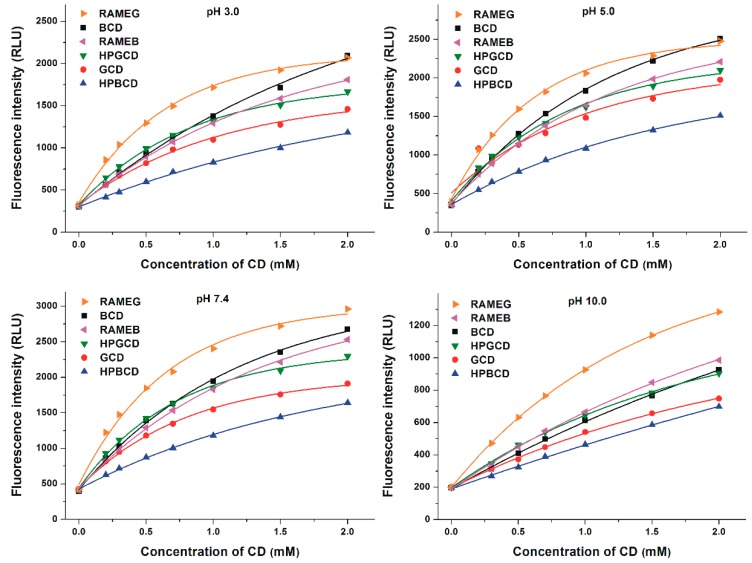
CD-induced increase in the fluorescence signal of Z14G (1 µM) in different buffers (0.05 M sodium phosphate, pH 3.0; 0.05 M sodium acetate, pH 5.0; 0.05 M sodium phosphate, pH 7.4; 0.05 M sodium borate, pH 10.0; λ_ex_ = 315 nm, λ_em_ = 455 nm).

The fluorescence emission intensities (λ_ex_ = 315 nm, λ_em_ = 455 nm) of Z14G in the presence of CDs are demonstrated in Figure 5, while the relative enhancement in the fluorescence of Z14G (*I*/*I*_0_; 1 μM mycotoxin + 2 mM CD) is represented in Table 1. RAMEB caused slightly weaker increase (except at pH 10.0) in the fluorescence of Z14G than BCD, while HPBCD showed a much weaker effect compared to the other β-CDs tested. However, both chemically modified γ-CDs proved to be better fluorescence enhancers than the native GCD: HPGCD and RAMEG induced slight and considerable increases in the fluorescence signal of Z14G, respectively (Table 1). Typically, RAMEG was the most suitable, and HPBCD was the least successful fluorescence enhancer; however, BCD exhibited similar effectiveness at pH 3.0 and 5.0 to RAMEG (Figure 5). Among the CDs tested, RAMEG resulted in the highest (7.1-fold) increase in the fluorescence signal of Z14G under weakly alkaline conditions (pH 7.4). BCD, HPBCD, GCD, and HPGCD induced the strongest relative increase in fluorescence at pH 5.0; while RAMEB and RAMEG were most effective at pH 3.0 and 7.4, respectively (Table 1). Each CD tested produced the weakest fluorescence enhancement at pH 10.0.

### 3.3. Binding Constants of Z14G-CD Complexes

**Figure 6:**
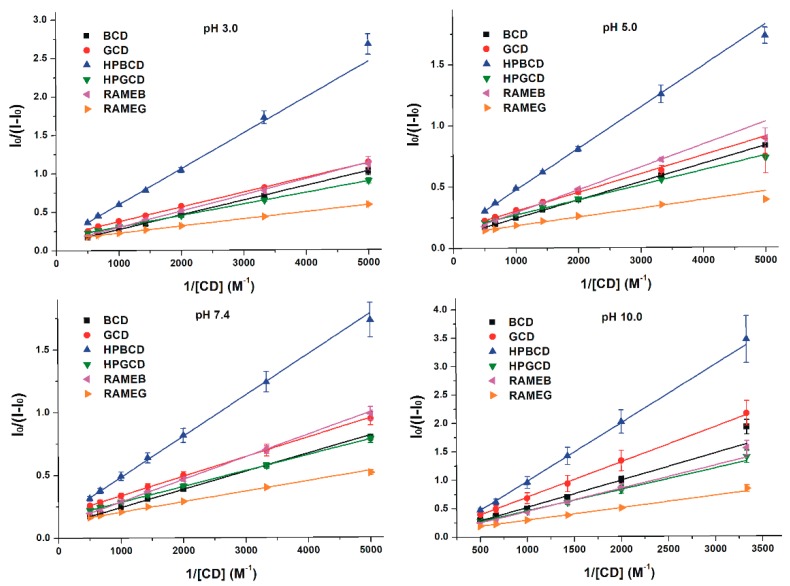
Benesi-Hildebrand plots of Z14G-CD complexes in different buffers (0.05 M sodium phosphate, pH 3.0; 0.05 M sodium acetate, pH 5.0; 0.05 M sodium phosphate, pH 7.4; 0.05 M sodium borate, pH 10.0; λ_ex_ = 315 nm, λ_em_ = 455 nm).

Binding constants (*K*, unit: L/mol) of Z14G-CD complexes were determined using the Benesi-Hildebrand equation (Equation (1)). Figure 6 demonstrates the Benesi-Hildebrand plots, which showed good correlation (R^2^ = 0.96–0.99) with the 1:1 stoichiometry model at each pH tested. As demonstrated in Table 2, Z14G formed stable complexes with both β- and γ-CDs, log*K* values were typically in the 2.5–3.3 range (except at pH 10.0). In the 3.0–7.4 pH range, γ-CDs formed more stable complexes with Z14G (log*K* = 3.0–3.3) than β-CDs (log*K* = 2.5–2.9). At pH 10.0, log*K* values were the lowest with regard to both β- and γ-CDs (log*K* = 1.9–2.4). The most stable Z14G-CD complex was formed with RAMEG at pH 5.0.

### 3.4. Molecular Modeling Studies

**Figure 7:**
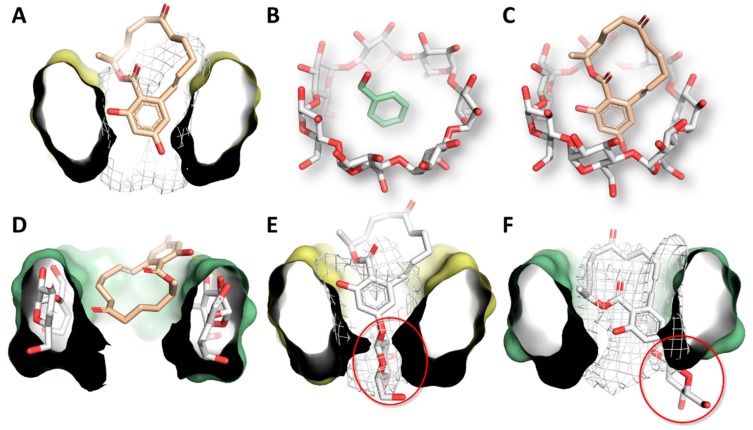
Representation of CD host–guest-type complexes. In most parts of the figure, CDs are represented as cut surfaces for the better clarity. (**A**) Representation of the calculated ZEN-BCD complex. The white mesh indicates the hydrophobic space of the BCD cavity; (**B**) Crystallographic coordinates of phenyl alcohol-BCD complex (CCDC accession code DEBGOG); (**C**) Calculated pose of ZEN within BCD; (**D**) Representation of the calculated ZEN-GCD complex. GCD is represented as sticks and cut surface. Yellow dashed lines indicate polar interaction; (**E**) Representation of the calculated Z14G-BCD complex. The white mesh indicates the hydrophobic space of BCD cavity while the red ring indicates the glucoside group of Z14G; (**F**) Representation of the calculated Z14G-GCD complex. The white mesh indicates the hydrophobic space of GCD cavity, while the red ring indicates the glycoside group of Z14G.

The molecular details of the interaction of Z14G within the native BCD and GCD were investigated and compared to those of ZEN using a molecular modeling approach. Concerning the ZEN-BCD complex, ZEN sank into the CD cavity with its aromatic ring. The calculated pose (179.4 HINT score units) was found to be well embedded within the hydrophobic environment of the BCD cavity (Figure 7A), retracing the crystallographic mode of interaction with phenyl alcohol (accession code of BCD-phenyl alcohol: DEBGOG) (Figure 7B,C). In this architecture of binding, no direct ZEN-CD polar contacts were found, and hydrophobic–hydrophobic interactions were thought to be the main contributors to the complex formation. Regarding the ZEN-GCD complex (64.7 HINT score units), in contrast to the pose observed within BCD, ZEN posed the aliphatic part within the cavity, exposing the aromatic ring to the solvent (Figure 7D). The hydroxyl group in position C_16_ was found to be engaged in polar contacts with the C_2_ and C_3_ hydroxyl groups of GCD sugars. Therefore, the establishment of polar interactions was found to be likely to concur with the complex formation.

Concerning the Z14G-BCD complex (101.3 HINT score units), Z14G posed the polar glucoside portion within the hydrophobic cavity of BCD (Figure 7E), causing hydrophobic–polar interactions that may explain the lower capability of Z14G to interact with BCD than with GCD. Indeed, regarding the Z14G-GCD complex (257.3 HINT score units), Z14G embedded the aromatic ring within the hydrophobic cavity of GCD while posing the glycoside moiety outside the cavity and exposing it to the solvent (Figure 7F). In this orientation, the glycoside moiety did not cause hydrophobic–polar interferences with the apolar cavity of GCD.

### 3.5. Extraction of Z14G from Aqueous Solution by BBP

**Figure 8:**
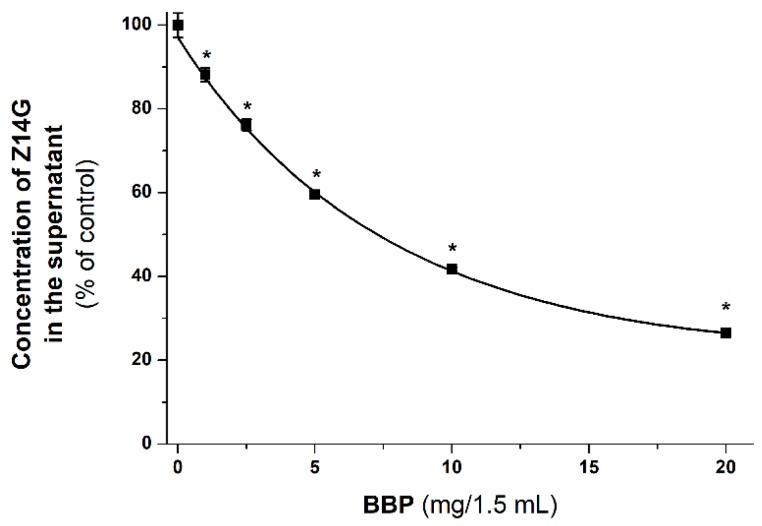
The decrease of Z14G (2 μM in 1.5 mL) content of the supernatant after 30 min incubation with increasing amounts of BBP (0.0, 1.0, 2.5, 5.0, 10.0, and 20.0 mg/1.5 mL) in 0.05 M sodium acetate buffer (pH 5.0; 25 °C; * *p* < 0.01).

Since BBP has been successfully applied to remove ZEN and zearalenols from aqueous solutions and/or from spiked corn beer samples [33], the ability of BBP to extract the masked mycotoxin Z14G from aqueous solution was also investigated. In BBP, the β-cyclodextrin polymer is attached to insoluble beads, therefore, the formed Z14G-BCD complex can be removed from the solution by sedimentation. To test the effect of BBP on the mycotoxin content of the solution, Z14G was incubated with increasing amounts of BBP in sodium acetate buffer (pH 5.0). In a concentration-dependent fashion, BBP considerably decreased the Z14G (2 μM) content of the aqueous solutions (Figure 8). Even 1.0 mg/1.5 mL of BBP significantly reduced the mycotoxin content; while 10.0 and 20.0 mg/1.5 mL of BBP removed approximately 60 and 75% of Z14G, respectively.

**Figure 9:**
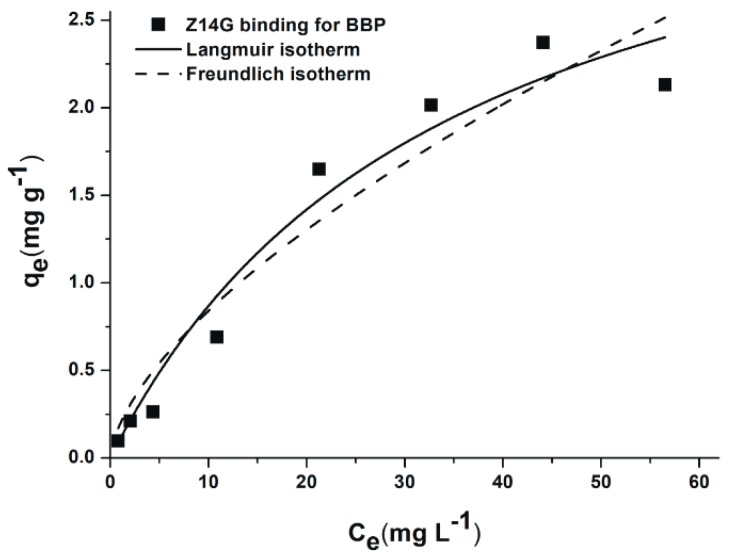
Langmuir (solid line) and Freundlich (dashed line) isotherms for the Z14G binding of BBP in 0.05 M sodium acetate buffer (pH 5.0; see further details in Section 2.4.).

To characterize quantitatively the mycotoxin-binding ability of BBP, increasing concentrations of Z14G were incubated with standard amount of BBP (see details in Section 2.4.). After incubation, the mycotoxin content of the supernatants was quantified (see in Section 2.4 and Section 2.5), and then the data were evaluated employing the Langmuir (Equation (2)) and Freundlich (Equation (3)) sorption isotherms. The data showed better fitting with the Langmuir (R^2^ = 0.96) than with the Freundlich (R^2^ = 0.92) isotherm (Figure 9). The Langmuir affinity constant (*K_L_*) was 0.0197 ± 0.006 L/mg and the *Q*_0_ value was 3.77 ± 0.49 mg/g. The Freundlich model indicates a 0.18 ± 0.01 (mg/g) × (L/mg) ^1/n^ Freundlich constant and a 0.64 ± 0.03 1/*n* value.

## 4. Discussion

Similar to ZEN and zearalenols [27,48], the masked mycotoxin Z14G also shows two excitation peaks at 275 and 315 nm (Figure 2). Z14G exerted fluorescence in the whole pH range tested (pH 3.0–10.0); however, its fluorescence spectra were markedly changed under alkaline conditions (pH 10.0). Regarding Z14G, only one of the phenolic hydroxyl groups (C_14_ and C_16_) of ZEN is conjugated with glucose. Therefore, Z14G can lose a proton and consequently it forms an anion at higher pH values. Thus, Z14G appears partly in ionized form at pH 10.0, resulting in the changes in its fluorescence excitation and emission spectra.

Similarly to our previous studies with ZEN and zearalenols [27,48], we recorded the emission spectra of Z14G-CD complexes using 315 nm excitation wavelength. In the presence of CDs, the fluorescence emission signal of Z14G strongly increased (Figure 3 and Figure 5). Since the applied CDs do not exert fluorescence, this observation suggests the formation of Z14G-CD complexes. The strong increase in the fluorescence of Z14G in the presence of CDs can be likely explained by the decreased quenching effects of solvent molecules. Usually, water molecules partly quench the fluorescence signal of aromatic fluorophores; therefore, the disruption of the hydration shell during the host–guest-type complex formation, as well as the less polar environment of Z14G in the CD cavity, results in the significant increase in the fluorescence signal of Z14G [23,25,30,45]. Similarly to ZEN [27], BCD induced a stronger increase in the fluorescence emission signal of Z14G than GCD (Figure 3). Concerning the ZEN-BCD complex, the calculated pose presented in this work (Figure 7) is in strong agreement with the NMR-derived model of ZEN-BCD complex proposed previously [31], supporting the geometrical reliability of the model. Keeping in mind that the inclusion direction can enhance or quench the fluorescence signal [46], the diverse calculated geometry of ZEN within BCD or GCD might partially explain the previously reported experimental results [27]. In particular, the deep inclusion of the fluorescent group (the aromatic ring of ZEN in this case) within the BCD cavity may reduce the capacity of the solvent molecules to absorb vibrational quanta, as previously described [46]. Therefore, BCDs protected the fluorescence emission of ZEN from the quenching effect of water molecules, during which the fluorescence signal is consequently enhanced. Conversely, the exposure of the aromatic ring to the solvent with widely spaced vibrational level (as in the case of ZEN-GCD complex in aqueous solution) allows the solvent to accept the large quantum of electronic energy, resulting in the higher quenching of the emission intensity [46]. In addition to the diverse orientation within BCD or GCD, the lower computational score of ZEN within GCD in comparison to BCD may indicate discrepancies in the favors of interaction between the two CDs, where the BCD is the most suitable to interact with ZEN. Nevertheless, a specific validation for the cavitand-ligand complex formation is still missing and further studies are needed to tune and validate the model for a more thorough quantitative comparison. 

The interaction of Z14G with native and chemically modified β-, and γ-CDs was tested in a wide pH range (pH 3.0–10.0). Approximately, a 3- to 7-fold CD-induced increase in the fluorescence of Z14G was observed (Table 1), which is significant but lower compared to ZEN (6- to 19-fold, pH 5.0) and zearalenols (2- to 26-fold, pH 5.0–10.0) [27,48]. The chemical modification (methyl or hydroxypropyl substitution) of CDs improved and decreased the CD-induced fluorescence enhancement regarding γ-CD and β-CD, respectively (Table 1). The poor elevation of the fluorescence signal of Z14G in the presence of CDs at pH 10.0 (Figure 4 and Figure 5) is likely resulted from the deprotonation of the mycotoxin, as it has been also reported regarding ZEN and zearalenols [27,48]. The same emission wavelength maximum of Z14G (455 nm) was observed in the presence of high CD concentrations, regardless the buffer used (Figure 4). It can be explained by the formation of the same Z14G-CD complexes. CDs form more stable complex with the non-ionized form of the mycotoxin; however, higher CD concentrations are necessary at pH 10.0, where most of the Z14G molecules are likely to occur in anionic form [27,48]. This hypothesis is also supported by the red shift in the fluorescence spectrum of Z14G during its interaction with CDs (Figure 4) and by the low binding constants of Z14G-CD complexes at pH 10.0 (Table 2). 

According to our data, the masked mycotoxin Z14G (log*K* = 2.8–3.3, at pH 5.0) formed less stable complexes with CDs compared to ZEN (log*K* = 3.8–4.8, at pH 5.0) [25,27,31]. Furthermore, Z14G forms more stable complexes with γ-CDs, while ZEN prefers β-CDs [27]. At pH 3.0–7.4, Z14G binds to GCD with approximately 2.5-fold higher affinity than to BCD (Table 2). The highly diverse arrangement of the glucose group regarding BCD and GCD may explain the higher affinity of Z14G toward the latter CD, wherein the hydrophobic-polar interferences were thought to be less pronounced than in the former (Figure 7). The chemical modifications (methyl and hydroxypropyl substitutions) of the native GCD slightly improved the stability of Z14G-γ-CD complexes; however, the same chemical modifications did not change, or slightly decreased, the binding constants of Z14G-β-CD complexes (Table 2). These observations highlight again the differences between the interactions of Z14G and ZEN with CDs, because methyl substitution of BCD strongly increased the stability of ZEN-CD complexes [27]. 

As our results demonstrate, the binding constants of Z14G complexes are barely affected by the pH under acidic and physiological conditions (pH 3.0–7.4). However, an approximately five-fold decrease in the binding constants was observed at pH 10.0 for each CD tested (Table 2). Under acidic and weakly alkaline conditions (pH 3.0, 5.0, and 7.4), Z14G likely appears in its nonionic form. However, at pH 10.0, several mycotoxin molecules become ionized (see spectral changes in Figure 2). Since the deprotonation of the hydroxyl group in C_16_ can affect the complex formation of Z14G with CDs, it is reasonable to hypothesize that CDs strongly prefer the nonionic Z14G vs. its anionic form.

As a general remark, the computational modeling reliably estimated the relative interaction of ZEN and Z14G with the two CDs, but it failed to estimate the absolute rank of affinity found experimentally [27]. In particular, the scores of Z14G were unexpectedly higher than those of ZEN, which recorded the best experimental affinity values within both the CDs, although the relative affinity of both ligands within each CD was correctly predicted. Keeping in mind that the HINT scoring is an expression of the sum of all the interatomic contributions [44], the overall score can be affected by the absolute dimension of ligands (i.e., by the total atoms count), as already shown for other scoring functions (in particular, the larger the molecule, the higher the value) [49]. The systems under analysis are relatively small, and they consist of a maximum of 234 atoms (e.g., the Z14G-GCD complex). The presence of glucose (24 atoms) accounts for nearly 10% of the total atom count of the complex and may reasonably introduce biases in comparing ligands with a relatively relevant difference in the total atom count. Therefore, the results of ZEN (45 atoms) cannot be quantitatively compared with those of Z14G (66 atoms; 46% bigger than ZEN). Although such a bias has been excluded for protein-ligand complex assessments, it still deserves further investigation regarding the assessment of cavitand-ligand complex formations. In the present form, our procedure proved to be reliable when comparing ZEN or Z14G within different CDs, but it failed to provide reliable quantitative intra-ligand comparison. Taken together, the computational results presented here can explain the diverse affinity of ZEN or Z14G with BCD and GCD. In particular, the different orientation of ZEN observed within the two CDs, along with the worse interaction within GCD (according to the scores recorded), may explain the stronger interaction with BCD found experimentally. On the other hand, our results can also explain the preferential interaction of Z14G with the GCD as the glucose moiety could be placed outside the cavity reducing the establishment of polar-hydrophobic interferences in comparison to those found for Z14G-BCD complex. 

Based on our results, BBP can greatly reduce the Z14G content of aqueous solution, in a concentration-dependent fashion. However, the removal of Z14G by BBP was less effective compared to ZEN. Under similar experimental conditions, BBP (20.0 mg/1.5 mL) removed approximately 75% and 90% of Z14G (Figure 8) and ZEN [33], respectively. The lower ability of BBP to extract Z14G is in a good agreement with the lower affinity of the masked mycotoxin (Table 2) towards BCD compared to ZEN [27]. It is reasonable to hypothesize that γ-CD polymers would be more effective in the removal of Z14G; however, BBP is the only CD bead polymer that was available for our studies. Furthermore, it was interesting to compare the Z14G-binding ability of BBP with our previous experiments regarding ZEN [33].

The sorption isotherms can quantitatively characterize of the mycotoxin-binding ability of BBP [35]. The Langmuir and Freundlich models were employed to investigate the interaction of Z14G with BBP (Figure 9). Based on our results, the Langmuir showed a better fitting vs. the Freundlich model. The Langmuir affinity constant of Z14G was significantly lower (0.0197 ± 0.006 L/mg) compared to ZEN (0.60 ± 0.25 L/mg) [33], which is in agreement with the lower binding affinity of BCD towards Z14G. The Freundlich constant of Z14G (0.18 ± 0.01 (mg/g) × (L/mg)^1/n^) was also lower compared to the *K_F_* value of ZEN (1.16 ± 0.07 (mg/g) × (L/mg)^1/n^) [33], again supporting the better ability of BBP to remove ZEN vs. Z14G. 

## 5. Conclusions

In summary, the interaction of Z14G with β- and γ-CDs, as well as with insoluble β-CD bead polymer, was examined. Despite the large hydrophilic glucose part of Z14G, it is able to form stable complexes with CDs; however, unlike ZEN, Z14G prefers the larger γ-CD cavity. CDs strongly increase the fluorescence signal of Z14G, and the methyl substitution of the native GCD can further increase both the fluorescence enhancement and the stability of formed complexes. BBP proved to be a suitable tool to decrease the Z14G content of aqueous solution, showing its ability to bind both the masked mycotoxin and the parent compound. Based on our observations, CD technology seems a promising tool to improve the fluorescence analytical detection of Z14G as well as to decrease the mycotoxin exposure through the removal of certain mycotoxins (e.g., Z14G, ZEN, and zearalenols) from aqueous solutions (including some beverages).

## Figures and Tables

**Table 1:** CD-induced relative increase in the fluorescence emission signal of Z14G (*I*/*I*_0_; 1 μM mycotoxin + 2 mM CD) in different buffers (λ_ex_ = 315 nm, λ_em_ = 455 nm).

pH	Relative Increase in the Fluorescence of Z14G (*I*/*I*_0_ ± SEM)					
	BCD	RAMEB	HPBCD	GCD	RAMEG	HPGCD
3.0	6.72 ± 0.12	6.23 ± 0.29	3.70 ± 0.09	5.03 ± 0.32	6.53 ± 0.08	5.38 ± 0.08
5.0	6.98 ± 0.19	6.16 ± 0.02	4.32 ± 0.03	5.49 ± 0.09	6.73 ± 0.22	5.79 ± 0.03
7.4	6.87 ± 0.10	5.93 ± 0.24	4.21 ± 0.21	4.92 ± 0.25	7.13 ± 0.10	5.53 ± 0.11
10.0	4.44 ± 0.14	4.99 ± 0.15	3.13 ± 0.14	3.62 ± 0.27	6.24 ± 0.17	4.37 ± 0.09

*BCD,* β-CD; *RAMEB,* randomly methylated β-CD; *HPBCD,* (2-hydroxypropyl)-β-CD; *GCD,* γ-CD; *RAMEG*, randomly methylated γ-CD; *HPGCD,* (2-hydroxypropyl)-γ-CD; *SEM*, standard error of the mean; buffers used: 0.05 M sodium phosphate, pH 3.0; 0.05 M sodium acetate, pH 5.0; 0.05 M sodium phosphate, pH 7.4; 0.05 M sodium borate, pH 10.0.

**Table 2:** Decimal logarithmic values of binding constants (*K*; unit: L/mol) of Z14G-CD complexes in different buffers.

pH	Log*K* (± SEM)					
	BCD	RAMEB	HPBCD	GCD	RAMEG	HPGCD
3.0	2.74 ± 0.05	2.64 ± 0.07	2.55 ± 0.06	2.99 ± 0.06	3.18 ± 0.03	3.03 ± 0.02
5.0	2.81 ± 0.02	2.81 ± 0.05	2.71 ± 0.06	3.03 ± 0.05	3.27 ± 0.02	3.13 ± 0.01
7.4	2.93 ± 0.01	2.85 ± 0.05	2.82 ± 0.03	3.14 ± 0.02	3.25 ± 0.01	3.13 ± 0.03
10.0	2.10 ± 0.07	2.21 ± 0.05	1.95 ± 0.05	2.30 ± 0.05	2.38 ± 0.07	2.41 ± 0.02

*BCD,* β-CD; *RAMEB,* randomly methylated β-CD; *HPBCD,* (2-hydroxypropyl)-β-CD; *GCD,* γ-CD; *RAMEG*, randomly methylated γ-CD; *HPGCD,* (2-hydroxypropyl)-γ-CD; *SEM*, standard error of the mean; buffers used: 0.05 M sodium phosphate, pH 3.0; 0.05 M sodium acetate, pH 5.0; 0.05 M sodium phosphate, pH 7.4; 0.05 M sodium borate, pH 10.0.
